# Attitudes toward free speech, content moderation, and punishment of misinformation spreaders during infectious disease outbreaks

**DOI:** 10.3389/fpubh.2026.1824585

**Published:** 2026-07-23

**Authors:** Jan Piasecki, Maria Zguda, Krzysztof Gerc, Natalia Segiet-Hojda, Michal Piksa, Rafal Ryguła

**Affiliations:** 1 Department of Bioethics and Health Psychology, Faculty of Health Sciences, Jagiellonian University Medical College, Krakow, Poland; 2 Department of Development and Health Psychology, Faculty of Management and Social Communication, Institute of Applied Psychology, Jagiellonian University, Krakow, Poland; 3 Doctoral School of Medical and Health Sciences, Jagiellonian University Medical College, Krakow, Poland; 4 Affective Cognitive Neuroscience Laboratory, Maj Institute of Pharmacology Polish Academy of Sciences, Krakow, Poland

**Keywords:** content moderation, public health ethics, ethics of content moderation, ethics of countering misinformation, social media, free speech, health education, infectious disease

## Abstract

**Introduction:**

During infectious disease outbreaks, the spread of false information online may undermine public health interventions. At the same time, free speech is a core value in democratic societies, and in the early stages of a novel pathogen there is a legitimate need for open discussion among scientists, policymakers, and the public to negotiate responses under conditions of uncertainty. Any restrictions on speech on social media should therefore reflect public attitudes and normative trade-offs. Currently, no standardized instrument exists to measure public attitudes toward free speech, content moderation, and punishment of misinformation spreaders on social media in the context of infectious disease outbreaks.

**Methods:**

In this study, we report the development and standardization of a 13-item questionnaire designed to assess these attitudes. The instrument was standardized in an online study conducted with a diverse sample of 1,002 adult, English-speaking participants in the United States recruited via Prolific.

**Results:**

Factor analysis identified three distinct dimensions capturing attitudes toward (1) free speech, (2) content moderation, and (3) punishment for misinformation spreaders.

**Discussion:**

The questionnaire provides a standardized tool for assessing public attitudes relevant to social media governance during health crises and may inform the design of public health communication strategies and evidence-informed policymaking.

## Introduction

During an infectious disease outbreak, social media may become a channel for false or misleading health information, which can, in turn, impede public health interventions (1). Since one of the main functions of free speech is the justification of laws and policies in a democratic society, censorship and restrictions on free speech on social media may, especially when there is no scientific consensus, undermine trust in science and public health authorities (2). Therefore, it is crucial for public health authorities to understand social media users’ preferences regarding free speech and speech governance policies in order to design information campaigns that enhance trust and promote reliable health information. We developed and standardized an instrument that measures social media users’ attitudes toward three factors in the context of an infectious disease outbreak: *free speech*, *content moderation*, and *punishment* for misinformation spreaders.

Initially, we aimed to develop a scale measuring attitudes toward different types of interventions designed to counter online misinformation. Our scoping review identified 15 types of interventions (3), which fell into three main categories proposed by Kozyreva et al.: boosting, nudging, and technocognition. However, our central research question was ethical and regulatory rather than psychological. We were concerned with the public health ethics of intervening in the information environment of social media in the context of an infectious disease outbreak.

For this reason, we did not classify the 15 intervention types according to their psychological mechanisms—whether they target cognitive and motivational competencies (boosting), shape behavior by altering choice architecture (nudging), or modify technological systems to enhance users’ cognitive capacities (technocognition). Instead, our point of departure lay in public health ethics and in the recognition of a moral tension between individual freedom—specifically free speech on social media—and public health goals, such as preventing the spread of misinformation, protecting from its harmful consequences, and promoting health (4).

The final three independent measurements—attitudes toward free speech, content moderation, and punishment for spreading misinformation—represent the key elements that can be meaningfully assessed during an infectious disease outbreak (see Table 1). Measuring attitudes toward free speech allows us to estimate the extent to which individuals or communities value free expression on social media, and how this valuation correlates with ideological and demographic factors. The other two measurements capture attitudes toward different forms of interference with free speech.

**Table 1:** Questionnaire.

No	Code	Question
1	freespch1	Every social media user has always a right to say whatever he/she wants.
2	freespch2	Every social media user has always a right to share any kind of information.
3	freespch3	Social media platforms should never censor any information created and shared by its users.
4	freespch4	Social media platforms should never label any information created by its users.
5	media_lit	Social media platforms may promote media literacy posts like this one: “Before you share, comment or like: make sure if this tweet is credible. Do you recognize the news organization/user that posted the story? Is information believable? Is this tweet politically motivated?”
6	health_edu	During the COVID-19 outbreak tweets from public health departments, World Health Organization and infectious disease experts may be visible to every social media user, regardless of whether a user follows the account or not.
7	counter_1	Posts that contain misinformation on an infectious diseases outbreak (as assessed by public health experts) may be tagged with a warning sign by social media platform (e.g., X, formerly Twitter, Facebook).
8	counter_2	Posts containing misinformation on an infectious diseases outbreak may be accompanied with corrections from fact-checking organizations or public health departments.
9	counter_3	Social media platforms should limit the possibility of reposting information about infectious diseases that is not supported by the current medical consensus.
10	counter_4	Social media platforms should have a right to temporarily ban users who spread misinformation on an infectious disease outbreak.
11	punish_5	Social media platforms should have a right to permanently ban users who spread misinformation on an infectious diseases outbreak (such as COVID-19).
12	punish_6	The government should have a right to demand social media platforms to ban users spreading health misinformation during an infectious disease outbreak (such as COVID-19).
13	punish_7	Spreading of health misinformation on social media should be a crime punishable with a fine

Content moderation represents a relatively soft form of speech regulation. It does not silence a speaker but places their expression in a different, unintended context. Importantly, this context is not generated through free exchange among equal participants, but is instead determined by experts who are presumed to have superior knowledge and who adjudicate what counts as information or misinformation on a given platform, and is enforced by social media platform mechanisms.

Punishment for spreading misinformation, by contrast, constitutes a much stricter form of speech control. It functions both as a deterrent and as a form of retribution against those who violate standards established by experts or platforms.

These three factors emerged empirically—from both qualitative data in the scoping review and quantitative data from the questionnaire. At the same time, they have clear theoretical justification. They reflect three distinct approaches to free speech on social media: a libertarian approach, in which speech should not be restricted (*free speech*); a liberal paternalistic approach, in which speech is moderated (*content moderation*); and a punitive approach, in which individuals face consequences for spreading misinformation (*punishment*) (5).

Our questionnaire contributes to empirical public health ethics and allows us to measure the strength of beliefs concerning free speech and misinformation on a standardized scale. This is crucial because attitudes toward free speech are not binary. Most people value free speech; however, they assign different moral weight to it. This means that the tool can be used to assess what kinds of value trade-offs may be acceptable for particular individuals and groups. For instance, if respondents do not score highly on the free speech scale, we may expect that there is room for negotiation and compromise, and that they may be more willing to accept certain limitations on speech. Conversely, a group may value free speech highly while at the same time strongly opposing any form of punishment or moderation. In such cases, it would be unwise to introduce harsh policies, as they could prove ineffective and potentially undermine trust in public health authorities and social media platforms, possibly leading to user migration. Moreover, public health policies or recommendations should be developed in light of public acceptance, and this tool can provide policymakers with reliable information about public attitudes.

Standardization of the scale allows for reliable and replicable comparisons across different groups and individuals, including cross-cultural and longitudinal comparisons. For instance, such a tool would enable us to assess attitudes toward *free speech*, *content moderation*, and *punishment* during an infectious disease outbreak. This is particularly important for policymakers, who may tailor public health interventions on the basis of empirical evidence.

However, the questionnaire does not address the actual health and medical education of participants. Individuals differ in their levels of health education and media literacy, as well as in their willingness to share false and true information online. These aspects were the focus of our previous studies, which distinguished four phenotypes of susceptibility to misinformation and factors that may underline it (6, 7).

For this paper, we adopt Nan et al.’s definition of health misinformation: “Health misinformation is a claim of fact about health promotion and/or disease control that is currently false due to its contradiction to expert consensus and/or best available evidence at the time, inaccurate due to its use of incomplete evidence, or unsubstantiated due to a lack of evidence” (8). This definition does not distinguish between intentional (disinformation) and unintentional (misinformation) falsehoods, nor does it engage with related concepts such as fake news or gossip (8, 9). However, it provides three objective reference points — contradiction, expert consensus, and best available evidence — which allow for the systematic assessment of any given information claim.

This measurement may also be used beyond the field of public health ethics, for example in research on broader political attitudes. In recent years, there has been increasing discussion of the concept of Left-Wing Authoritarianism (10, 11). This tool could be used to explore and further differentiate this concept.

Here, we present a tool that was developed and standardized for a US, English-speaking population. Statistical analysis reveals that the questionnaire can be used to measure users’ attitudes toward three different factors: free speech, content moderation, and punishment for those who spread misinformation in the context of an infectious disease outbreak.

## Methods

### Procedure

We have been guided by the framework for scale development in health, social and behavioral research, while adopting the procedure to the aims and constrains of the present study (12).

*Item development*. Firstly, we identified the domain of our interest, mainly, already mentioned ethical conflict between freedom of speech on social media and need for limiting of harmful health misinformation.

Next, we hypothesized different attitudinal constructs in the domain of interests. We thought that social media users may have different attitudes towards freedom of speech and that social media deployed interventions aiming at altering users’ behavior concerning health information differ in respect to the level of interfering into personal freedom. Therefore, it was hypothesized that strong support for free speech may also entail tolerance of less interfering counter misinformation interventions.

We decided to develop a questionnaire using 7-point Likert scale (Strongly disagree – Neither agree nor disagree – Strongly agree) to measure attitudes toward free speech and different counter misinformation interventions. Likert-type scales enable the quantification of subjective attitudes and preferences by capturing graded levels of agreement or disagreement. Although individual Likert items produce ordinal data and may not satisfy normality assumptions, composite scales formed from multiple items often approximate interval-level measurement and can, under appropriate conditions, be analyzed using parametric methods. Thus, Likert scales are used for assessing attitudes toward policies, values, and social issues (13).

Generation of items for the scale consisted of several steps. Free speech items were initially generated based on review literature on that topic. Misinformation countering items were developed based on a scoping review of different interventions countering health misinformation (3).

In the next step, a preliminary set of items, intended to capture attitudes toward free speech and a variety of interventions (promoting health and media literacy, curbing the spread of false health information, punishing and deterring potential and actual spreaders of health misinformation on Twitter in the context of the COVID-19 pandemic), was informally reviewed for accuracy, clarity, and linguistic correctness. This review incorporated feedback from #webimmunization project research members and the administrative team, as well as social media users. The final version of the COVID-19-related questionnaire included 13 items (see Supplementary material S1).

*Scale development.* The development of a scale requires statistical analyses to assess the internal consistency and reliability of its items. Commonly used methods include Cronbach’s alpha and factor analysis, which provide evidence about whether the scale reliably measures the intended theoretical constructs (14).

In the initial study, the COVID-19–related questionnaire was administered to 2,997 adult American users of Twitter (now X) as part of a larger study on susceptibility to misinformation. Internal consistency was assessed using Cronbach’s alpha, and exploratory factor analysis was conducted to examine the structure of the scale. The results indicated that the questionnaire was reliable and supported the validity of the underlying theoretical constructs (see Supplementary material S1).

In that study, the questionnaire was intended to capture potential differences in political views, susceptibility to misinformation, and attitudes toward free speech policies. The data analysis indicated that the questionnaire can be used as a reliable instrument for measuring attitudes toward free speech policies. However, the questionnaire needed to be adjusted to the current epidemiological context.

The recent study aimed to standardize a revised questionnaire measuring attitudes toward free speech and policies countering online misinformation (see Table 1). The questionnaire was administered to 1,044 American adult citizens recruited via Prolific (15), a UK-based internet platform that connects researchers with a vetted participant pool. We requested Prolific’s “standard sample,” which approximates the US adult population in terms of age, gender, and ethnicity, though it is not a probability sample. Participants were selected based on their current residence in the United States. The study was conducted in English, and participants were compensated at a rate of £8.97/h, in compliance with UK labor laws. Data were collected on February 6, 2025, using Qualtrics for survey administration. All participants provided informed consent via Prolific after reviewing study information.

During the survey we did not collected personal data and all raw data are accessible in an open Jagiellonian University Repository. Before commencing the research we received approval from the Research Ethics Committee at the Jagiellonian University Medical College (No. 1180043.1.504.2024, January 14 2025).

### Participants

We recruited 1,044 adult American citizens and included 1,002 participants in our analysis. We excluded those who failed one of two attention checks (*n* = 39; “Please mark Somewhat disagree just so we can check if you are paying attention” and “Please mark Neither agree nor disagree just so we can check if you are paying attention”) or who did not complete the questionnaire (*n* = 3).

The median age was 39 years. Of the sample, 44% were male, 55% female, 1% third gender/non-binary/other, and <1% preferred not to disclose their gender identity.

In terms of ethnicity, 74% were White, 13% Black, 6% Asian, 1% American Indian or Alaska Native, <1% Native Hawaiian or Pacific Islander, and 3% identified as multiple races. According to the 2025 US census, the population was 74.8% White alone and 13.7% Black alone (16), indicating that our sample largely mirrored the population. One exception was the Hispanic or Latino group, which was absent in our sample despite representing 20% of the US population. This may be due to the study being conducted in English, which could have discouraged participation from individuals whose primary language is Spanish.

Regarding political affiliation, 30% of participants identified as Republicans, 35% as Democrats, and 32% as Independents. According to the PEW Research Fact Sheet in 2025, 31% of U. S. adults identified as Republican, 28% as Democrat, and 41% as Independent or “something else” (17). Thus, our survey participants’ distribution was broadly comparable to the US population.

Detailed demographic information of participants included in the analysis is presented in Table 2.

**Table 2:** Demographic data for participants included in analysis.

Characteristic	*N*	%
All participants	1,044	100%
Included in analysis	1,002	95.98%
Excluded from analysis	42	4.02%
Incomplete data	3	0.29%
Failed attention check	39	3.74%
Age		
Median	39 (IQR = 18)	
Mean	41 (SD = 12.9)	
Gender		
Male	437	43.61%
Female	551	54.99%
Third gender/nonbinary/other	12	1.20%
Prefer not to say	2	0.20%
Employment		
Working full-time	577	57.58%
Working part-time	174	17.37%
Unemployed or looking for work	60	5.99%
A homemaker or stay-at-home parent	82	8.18%
Student	26	2.59%
Retired	60	5.99%
Other	23	2.30%
Marital status		
Married	480	47.90%
Living with a partner	120	11.98%
Widowed	20	2.00%
Divorced/Separated	89	8.88%
Never been married	293	29.24%
Education		
Less than high school degree	12	1.20%
High school graduate	121	12.08%
Some college but not degree	166	16.57%
Associate degree in college (2-year)	107	10.68%
Bachelor’s degree in college (4-year)	406	40.52%
Master’s degree	187	18.66%
Doctoral degree	3	0.30%
Political affiliation		
Republican	295	29.44%
Democrat	356	35.53%
Independent	319	31.84%
Something else	32	3.19%
Ethnicity		
White	745	74.35%
Black or African American	131	13.07%
American Indian or Alaska Native	6	0.60%
Asian	63	6.29%
Native Hawaiian or Pacific Islander	1	0.10%
Other	15	1.50%
Prefer not to say	7	0.70%
Multiple races	34	3.39%

### Statistical methods

We used standard statistical tools to assess the reliability of the questionnaire as a whole and of selected items. Analyses were conducted in JASP software, which allows for complex and detailed modeling. A scree plot, factor analysis, and tests of various data-fitting and factor-structuring models were performed.

To assess the instrument and optimally extract factors independently of prior assumptions—given that the questionnaire was in its pilot version—an exploratory factor analysis was carried out.

We calculated Cronbach’s alpha (*α*) for the questionnaire and for selected items, and used chi-square tests to examine relationships between factors based on internal correlations among items. Although Cronbach’s α is the most widely used metric for evaluating internal consistency, it has limitations; for example, it inherently assumes one-dimensionality (14, 18). To address these limitations, we implemented factor analysis to scrutinize the actual structure of the data. Additionally, to further verify internal consistency, we calculated McDonald’s omega (*ω*), an alternative to α that more effectively handles congeneric and tau-equivalent models, where items may measure related but distinct aspects of a construct (14, 18).

## Results

Cronbach’s alpha for the full questionnaire—using the identified factor structure and retaining all items—was 0.724, and McDonald’s omega (ω) was 0.722, indicating an acceptable level of reliability. Detailed calculations for the full questionnaire and each of the three factors are presented in Table 3.

**Table 3:** Internal consistency estimates (McDonald’s ω and Cronbach’s α) for the complete questionnaire presented in Table 1.

Estimate	McDonald’s *ω*	Cronbach’s *α*
Point estimate	**0.722**	**0.724**
95% CI lower bound	0.708	0.708
95% CI upper bound	0.737	0.739

Bold values indicate the main values of interest.

**Figure 1:**
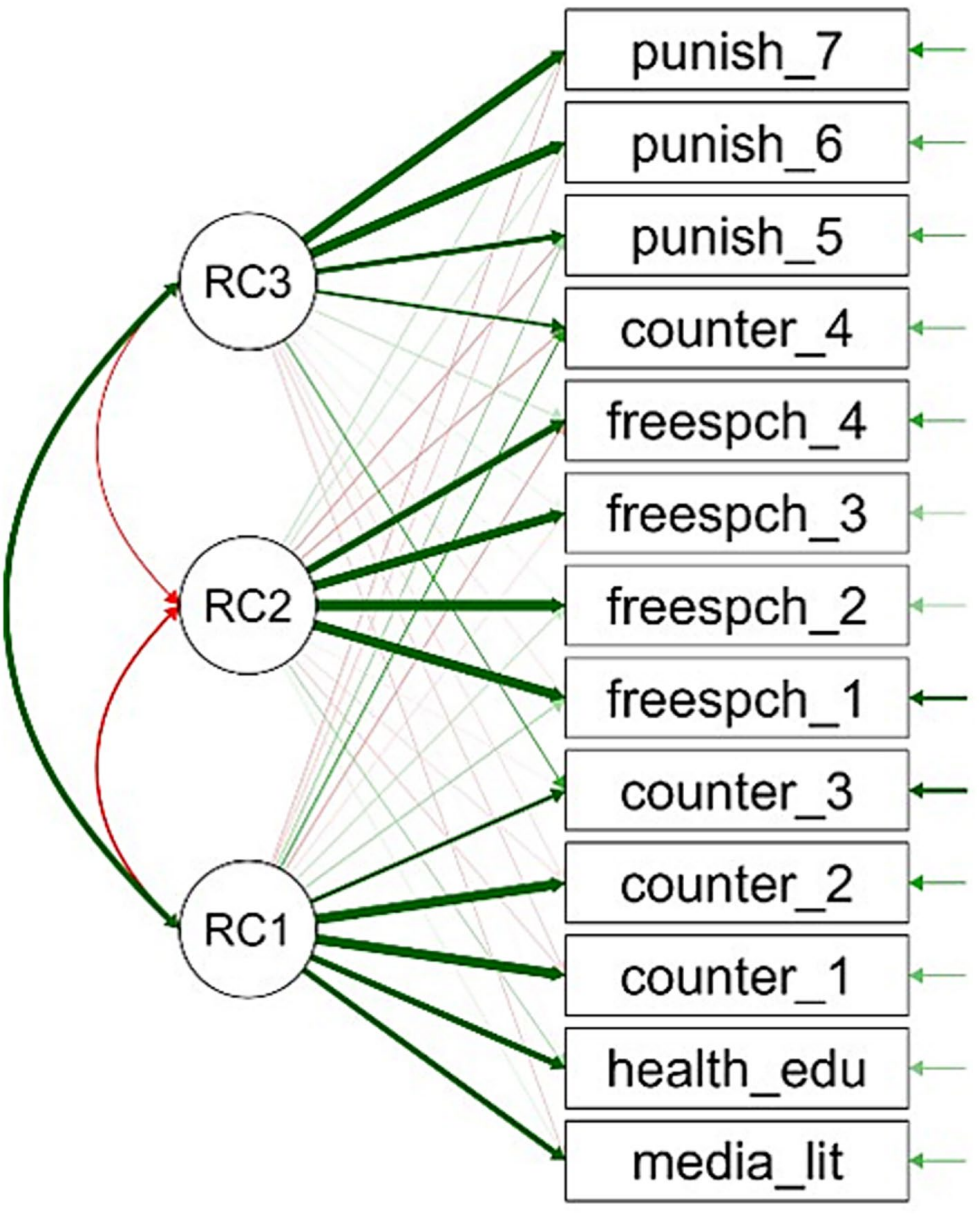
Distribution of questionnaire factors with items assigned based on the analysis results.

Originally, we hypothesized that the questionnaire would capture five distinct attitudes toward policies controlling speech on social media. Questions Q1–Q4 were intended to measure attitudes toward free speech (Figure 1, tags “freespch”), Q5 toward media literacy (Figure 1, tag “media_lit”), and Q6 toward health education (Figure 1, tag “health_edu”). Questions Q7–Q9 addressed technological or cognitive measures countering misinformation, such as warning tags, debunking, or limiting reposting (Figure 1, tags “counter”), while Q10–Q13 targeted attitudes toward punishment for users spreading misinformation (Figure 1, tags “punish”).

However, exploratory analysis of the statistical models revealed a different structure than originally assumed. Three factors—RC1, RC2, and RC3—emerged from inter-item correlations (Figure 1): RC1 (Q5–Q8, content moderation), RC2 (Q1–Q4, free speech), and RC3 (Q9–Q13, punishment for misinformation spreaders). These three subscales can therefore be used independently.

Reliability for each subscale was strong: Cronbach’s alpha for *content moderation* was 0.821 (Tables 4, 5), for *free speech* 0.913 (Tables 6, 7), and for punishment 0.881 (Tables 8, 9). These results indicate high internal consistency across the subscales.

**Table 4:** Internal consistency estimates (McDonald’s ω and Cronbach’s *α*) for factor 1 (RC1: content moderation subscale: items Q5–Q9).

Estimate	McDonald’s *ω*	Cronbach’s *α*
Point estimate	0.828	**0.821**
95% CI lower bound	0.818	0.810
95% CI upper bound	0.838	0.831

Bold values indicate the main values of interest.

**Table 5:** Internal consistency estimates for factor 1 following item deletion.

Item	Cronbach’s α for the Factor 1
Q5: media_lit	0.807
Q6: health_edu	0.793
Q7: counter_1	0.723
Q9: counter_3	0.767

Column 1 lists the omitted item; Column 2 reports Cronbach’s α for the remaining items.

**Table 6:** Internal consistency estimates (Cronbach’s α) for factor 2 (RC2: free speech subscale; items Q1–Q4).

Estimate	Cronbach’s α
Point estimate	**0.913**
95% CI lower bound	0.908
95% CI upper bound	0.918

Bold values indicate the main values of interest.

**Table 7:** Internal consistency estimates for factor 2 following item deletion.

Item	Cronbach’s α for the factor 2
Q1: freespch_1	0.891
Q2: freespch_2	0.875
Q3: freespch_3	0.876
Q4: freespch_4	0.905

Column 1 lists the omitted item; Column 2 reports Cronbach’s α for the remaining items.

**Table 8:** Internal consistency estimates (McDonald’s ω and Cronbach’s α) for factor 3 (RC3: punishment subscale; items Q1–Q4).

Estimate	McDonald’s ω	Cronbach’s α
Point estimate	0.879	**0.881**
95% CI lower bound	0.871	0.874
95% CI upper bound	0.886	0.888

Bold values indicate the main values of interest.

**Table 9:** Internal consistency estimates for factor 3 following item deletion.

Item	Cronbach’s α for the Factor 3
Q10: counter_4	0.846
Q11: punish_5	0.829
Q12: punish_6	0.846
Q13: punish_7	0.868

Column 1 lists the omitted item; Column 2 reports Cronbach’s α for the remaining items.

Another round of statistical analysis was conducted to further validate the instrument. To evaluate the initially proposed factor model, an exploratory analysis was repeated to determine whether the scree plot would again identify three factors and whether the current model would organize them similarly to the first study. Three factors were once again extracted, although one item was reassigned to a different factor. Additionally, a confirmatory factor analysis of the original model confirmed good model fit; the software extracted the factors in a different order (RC1 and RC2 were swapped), which does not affect the overall positive evaluation of the questionnaire or its reliability. Detailed calculations are provided in the Supplementary material S2.

## Discussion

Our analysis confirms that the questionnaire can be used to measure three distinct factors in the context of social media during an infectious disease outbreak: RC1 (Q5–Q9) – attitudes toward *content moderation*; RC2 (Q1–Q4) – attitudes toward *free speech* on social media; and RC3 (Q10–Q13) – attitudes toward *punishment*. These factors were refined based on statistical analyses and inter-item correlations.

The main adjustment relative to the initial assumptions was that temporary banning a user is now interpreted as a form of punishment rather than a policy to manage misinformation. Initially, we focused on the fact that the goal of a temporary ban of a user is to limit the spread of false information from that account. However, this policy can also be perceived as a form of punishment by a user who is banned and cannot use their account to publish information or even to access information on a social media platform. Additionally, the statistical analysis revealed a stronger correlation between a temporary ban and other items belonging to the category of punishment for misinformation spreaders. Therefore, we decided to move this item into that category. Consequently, we received two consistent categories: content moderation and punishment for spreaders of misinformation. Interventions such as promoting media literacy and health literacy, tagging posts with warnings, and attaching debunking or corrections to posts containing misinformation can be grouped into a single category, which we call content moderation on social media. These policies do not restrict users’ free speech, but they alter the social media experience by promoting certain values and introducing independent assessments of information not chosen by the user. In this sense, content moderation can be understood as a form of paternalism.

The questionnaire enables measurement of three aspects of attitudes toward speech control on social media during an infectious disease outbreak: (1) attitudes toward free speech, (2) attitudes toward content moderation, and (3) attitudes toward punishment of misinformation spreaders. It is reasonable to expect that the first aspect is negatively correlated with the other two. Individuals who strongly support free speech (high scores on factor 1) may resist interventions in social media content, while both content moderation and punishment can be interpreted as forms of paternalism or censorship. Conversely, individuals who strongly support interventions to enhance information quality and punishment for misinformation spreaders are unlikely to score highly on free speech policies.

Thus, the overall questionnaire score should not be used as a single indicator of attitudes. Instead, the instrument provides three independent scores—or, broadly, two composite scores: free speech attitudes (Q1–Q4) and attitudes toward content moderation (Q5–Q9), and attitudes toward punishment for misinformation spreaders (Q10–Q13). In a broader sense, these scores could serve as proxies for underlying dispositions, such as tolerance for risk versus desire for safety, though this requires further research. Free speech may reflect higher risk tolerance, whereas support for speech governance may indicate a stronger preference for safety in public life.

Potential applications of the scale include comparing individuals’ and groups’ attitudes toward free speech and interventions that limit misinformation during a health emergency. Understanding these attitudes can inform the design of speech governance policies or public information campaigns. For instance, campaigns targeting individuals with high support for free speech and low support for interventions may require different strategies than those directed at individuals who accept limits on free speech and support social media governance. Moreover, the scale has the potential to be used to monitor how attitudes toward free speech, content moderation, and punishment change over the course of an infectious disease outbreak. However, we did not test the responsiveness of the scale in the current study; this remains a task for future research.

Moreover, individual levels of health and media literacy may be related not only to susceptibility to misinformation (6) but also to attitudes toward *free speech*, *content moderation*, and *punishment*. This possibility opens new avenues for research using the questionnaire.

However, there are also some limitations and directions for future developments in this area. There some limitations of this tool that also can lead to future research and developments.

The questionnaire was originally devised during the COVID-19 pandemic and its newer version is more general, but still measures attitudes towards free speech, content moderation, and punishment for spread of misinformation in the context of infectious diseases. However, one can imagine that other scales more general and more specific could be developed based on this narrowly focused version. One may think about free speech attitudes in the context of scientific consensus and public health policies, such as vaccination mandates or general attitudes toward free speech on social media and health misinformation.

The questionnaire does not explicitly conceptualize or define misinformation or health expertise; however, both terms are present in the questionnaire items. At the same time, the questionnaire tacitly assumes the previously mentioned definition of misinformation provided by Nan et al., which itself refers to the concepts of misinformation and health expertise. Item 6 (health_edu) mentions public health departments, the World Health Organization, and infectious disease experts, assuming that these are credible sources of information, while item 7 (counter_1) again refers to public health experts who help social media platforms identify health misinformation (Table 1).

A skeptic may raise two main objections to this approach. First, the skeptic may argue that if there is no expert consensus based on evidence, we cannot define what constitutes health misinformation. The second argument is that we do not have a moral right to limit someone’s speech without a justified reason. Moreover, the most important element of justification for limiting speech in the context of a pandemic is public health, which can only be prioritized when there is sufficient evidence—taking us back to square one, where it has already been stated that there is no such evidence.

In response to these objections, it must be admitted that during a health emergency—such as a pandemic caused by a novel virus like SARS-CoV-2—high uncertainty and a lack of robust evidence often prevent a scientific consensus among experts. Furthermore, some experts’ opinions may be discarded not on an evidential basis, but because of bias and differing value judgments (19, 20). In such situations, decision-makers, politicians, and scientists may present certain ideas or policy recommendations as established science, potentially labeling dissenters or skeptics as spreaders of misinformation (20).

However, a lack of expert consensus or evidence and unjustified censorship are two distinct issues. From the fact that certain scientific and public institutions fail to recognize genuine disagreement between health experts—or a lack of scientific evidence in certain aspects of health science—it does not follow that there is no false and potentially harmful health misinformation where all experts *do* agree (e.g., drinking alcohol to prevent a viral infection). Right now, there is a broad discussion on rebuilding trust in science, appreciating expert disagreement, and ensuring transparency in public communication. Thus, it can be reiterated that there are experts and there is scientific consensus; however, its scope could be narrower than it is sometimes presented. Besides, possible interventions targeting misinformation and its spreaders should reflect genuine consensus and the state of the art, rather than the opinions of a few well-established experts.

In response to the skeptic’s second objection, it must be admitted that different interventions into the information sphere are at odds with freedom of speech and, more broadly, the principle of respect for autonomy. One can argue, for instance, that inoculation strategies or nudging—which operate without public awareness or deliberative scrutiny—aim to shape how people process and accept information, and in doing so, undermine both science and democracy (21). They conflict with science because scientific progress depends on open contestation and competition among claims. They also conflict with democratic governance, where transparency and open discourse in free public deliberation enable the negotiation of the common good (21).

However, new media technologies and the rapid spread of false and harmful misinformation may sometimes require a more paternalistic approach. Information targeting false claims on social media can be considered a public health intervention. Childress et al. argue that a public health intervention is justified when it is effective in protecting or improving public health, and when the provided benefits are proportional to the infringed individual autonomy, necessary, least infringing, and publicly justified in public discourse. If we consider the case of Iran, where a swiftly spreading medical myth that alcohol prevents COVID-19 infection led to alcohol poisoning and hundreds of deaths, it seems that many different interventions limiting the online spread of this myth would be justified in accordance with those conditions. Finally, it can be added that the questionnaire discussed also public opinion surveys about the ethical acceptability of different intervention measures.

Another limitation of the study is that we did not follow the formalized requirements of the COnsensus-based Standards for the selection of health Measurement INstruments (COSMIN) (22), which require the calculation of a content validity index (CVI), item-level content validity index (I-CVI), or scale-level content validity index (S-CVI). One could argue that the strong internal consistency results reported earlier are insufficient to prove the content validity and comprehensiveness of the target domain. However, item generation was grounded in a systematic scoping review conducted in accordance with the PRISMA Extension for Scoping Reviews (3, 23) to ensure domain comprehensiveness. Furthermore, to evaluate item relevance and comprehensibility, we conducted a qualitative pre-testing phase with both the research and administrative teams, as well as a group of regular social media users. These users were presented with the questionnaire and provided written feedback regarding their understanding of the questions alongside general comments.

## Conclusion

Social media pose a challenge to free speech in democratic societies and to public health. We developed a questionnaire intended to measure attitudes toward different speech policies on social media. Our aim was to capture attitudes toward free speech, media literacy, health education, countering misinformation, and punishing the spreaders of misinformation. Statistical analysis allowed us to distinguish three factors measuring attitudes toward *free speech*, *content moderation*, and *punishment for misinformation spreaders*. Attitudes toward free speech correlate negatively with the other two factors. Therefore, the questionnaire does not yield a single score for an individual but instead provides three separate measures, which can be used to assess individual and group attitudes toward free speech policies on social media during an infectious disease outbreak.

## Data Availability

The datasets presented in this study can be found in online repositories. The names of the repository/repositories and accession number(s) can be found in the article/Supplementary material.
