# Supplementary material for: Attitudes toward free speech, content moderation, and punishment of misinformation spreaders during infectious disease outbreaks
Source: Front Public Health. 2026 Jul 23;14:1824585. doi: 10.3389/fpubh.2026.1824585 (PMC13443021; doi:10.3389/fpubh.2026.1824585)

Results

Exploratory Factor Analysis

Chi-squared Test

	Value	df	p
Model	647.777	42	< .001

Factor Loadings

	Factor 1	Factor 2	Factor 3	Uniqueness
Q12	0.934			0.278
Q13	0.856			0.389
Q11	0.766			0.257
Q10	0.686			0.190
Q9	0.512			0.380
Q7		0.921		0.240
Q8		0.913		0.256
Q6		0.548		0.688
Q5		0.528		0.702
Q2			0.912	0.267
Q1			0.862	0.359
Q3			0.623	0.482
Q4			0.538	0.545

Note. Applied rotation method is promax.

Factor Characteristics

	Unrotated solution			Rotated solution		
	SumSq. Loadings	Proportion var.	Cumulative	SumSq. Loadings	Proportion var.	Cumulative
Factor 1	5.510	0.424	0.424	3.049	0.235	0.235
Factor 2	1.573	0.121	0.545	2.665	0.205	0.440
Factor 3	0.885	0.068	0.613	2.254	0.173	0.613

Factor Correlations

	Factor 1	Factor 2	Factor 3
Factor 1	1.000	0.663	−0.400
Factor 2	0.663	1.000	−0.447
Factor 3	−0.400	−0.447	1.000

Additional fit indices

RMSEA	RMSEA 90% confidence	SRMR	TLI	CFI	BIC
0.118	0.11 - 0.126	0.033	0.861	0.926	356.287

Scree plot

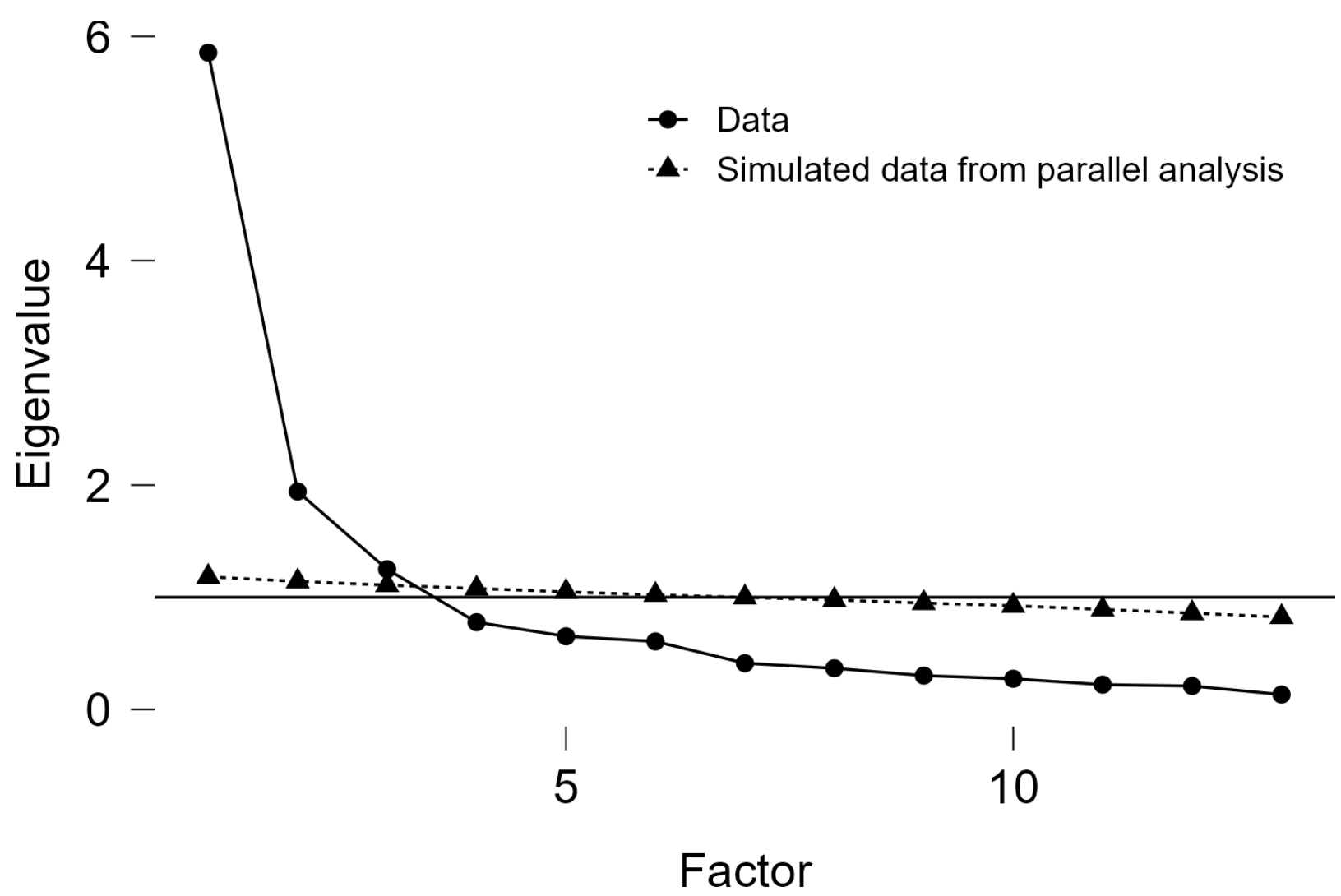

6
4
2
0
Eigenvalue
Data
Simulated data from parallel analysis
5
10
Factor

Confirmatory Factor Analysis

Model fit

Chi-square test

Model	X^2	df	p
Baseline model	76513.832	78	
Factor model	1445.972	62	< .001

Additional fit measures

Fit indices

Index	Value
Comparative Fit Index (CFI)	0.982
Tucker-Lewis Index (TLI)	0.977
Bentler-Bonett Non-normed Fit Index (NNFI)	0.977
Bentler-Bonett Normed Fit Index (NFI)	0.981
Parsimony Normed Fit Index (PNFI)	0.780
Bollen's Relative Fit Index (RFI)	0.976
Bollen's Incremental Fit Index (IFI)	0.982
Relative Noncentrality Index (RNI)	0.982

Information criteria

Information criteria are only available with ML-type estimators

Akaike (AIC)	
Bayesian (BIC)	
Sample-size adjusted Bayesian (SSABIC)	

Other fit measures

Metric	Value
Root mean square error of approximation (RMSEA)	0.147
RMSEA 90% CI lower bound	0.141
RMSEA 90% CI upper bound	0.154
RMSEA p-value	6.828×10^{-14}
Standardized root mean square residual (SRMR)	0.082
Hoelter's critical N (α = .05)	59.082
Hoelter's critical N (α = .01)	65.806
Goodness of fit index (GFI)	0.986
McDonald fit index (MFI)	0.511
Expected cross validation index (ECVI)	

Kaiser-Meyer-Olkin (KMO) test

Indicator	MSA
Q1	0.740
Q2	0.748
Q3	0.862
Q4	0.865
Q5	0.965
Q6	0.941
Q7	0.874
Q8	0.873
Q9	0.953
Q10	0.889
Q11	0.886
Q12	0.903
Q13	0.919
Overall	0.881

Bartlett's test of sphericity

X^2	df	p
10015.716	78	< .001

R-Squared

	R^2
Q1	0.677
Q2	0.738
Q3	0.670
Q4	0.624
Q5	0.320
Q6	0.309
Q7	0.778
Q8	0.764
Q9	0.763
Q10	0.942
Q11	0.830
Q12	0.680
Q13	0.614

Parameter estimates

Factor loadings

Factor	Indicator	Symbol	Estimate	Std. Error	z-value	p	95% Confidence Interval	
							Lower	Upper
Factor 1	Q1	λ11	0.823	0.011	71.826	< .001	0.800	0.845
	Q2	λ12	0.859	0.012	74.000	< .001	0.836	0.882
	Q3	λ13	0.818	0.012	69.909	< .001	0.795	0.841
	Q4	λ14	0.790	0.012	65.318	< .001	0.766	0.814
Factor 2	Q5	λ21	0.565	0.011	52.091	< .001	0.544	0.587
	Q6	λ22	0.556	0.011	52.627	< .001	0.536	0.577
	Q7	λ23	0.882	0.009	100.846	< .001	0.865	0.899
	Q8	λ24	0.874	0.009	99.088	< .001	0.857	0.891
	Q9	λ25	0.874	0.009	92.032	< .001	0.855	0.892
Factor 3	Q10	λ31	0.970	0.007	131.846	< .001	0.956	0.985
	Q11	λ32	0.911	0.007	129.237	< .001	0.897	0.925
	Q12	λ33	0.824	0.008	104.242	< .001	0.809	0.840
	Q13	λ34	0.783	0.009	90.312	< .001	0.766	0.800

Factor variances

Factor	Estimate	Std. Error	z-value	p	95% Confidence Interval	
					Lower	Upper
Factor 1	1.000	0.000			1.000	1.000
Factor 2	1.000	0.000			1.000	1.000
Factor 3	1.000	0.000			1.000	1.000

Factor Covariances

			Estimate	Std. Error	z-value	p	95% Confidence Interval	
							Lower	Upper
Factor 1	↔	Factor 2	−0.463	0.010	−47.748	< .001	−0.482	−0.444
Factor 1	↔	Factor 3	−0.454	0.009	−48.743	< .001	−0.472	−0.435
Factor 2	↔	Factor 3	0.785	0.008	97.147	< .001	0.769	0.801

Residual variances

Indicator	Estimate	Std. Error	z-value	p	95% Confidence Interval	
					Lower	Upper
Q1	0.323	0.000			0.323	0.323
Q2	0.262	0.000			0.262	0.262
Q3	0.330	0.000			0.330	0.330
Q4	0.376	0.000			0.376	0.376
Q5	0.680	0.000			0.680	0.680
Q6	0.691	0.000			0.691	0.691
Q7	0.222	0.000			0.222	0.222
Q8	0.236	0.000			0.236	0.236
Q9	0.237	0.000			0.237	0.237
Q10	0.058	0.000			0.058	0.058
Q11	0.170	0.000			0.170	0.170
Q12	0.320	0.000			0.320	0.320
Q13	0.386	0.000			0.386	0.386

Implied covariance matrix

Q1	Q2	Q3	Q4	Q5	Q6	Q7	Q8	Q9	Q10	Q11	Q12	Q13
1.000												
0.707	1.000											
0.673	0.703	1.000										
0.650	0.679	0.647	1.000									
−0.215	−0.225	−0.214	−0.207	1.000								
−0.212	−0.221	−0.211	−0.203	0.314	1.000							
−0.336	−0.351	−0.334	−0.322	0.498	0.491	1.000						
−0.333	−0.348	−0.331	−0.320	0.494	0.486	0.771	1.000					
−0.333	−0.347	−0.331	−0.319	0.494	0.486	0.770	0.764	1.000				
−0.362	−0.378	−0.360	−0.348	0.431	0.424	0.672	0.666	0.665	1.000			
−0.340	−0.355	−0.338	−0.327	0.404	0.398	0.631	0.625	0.625	0.884	1.000		
−0.308	−0.321	−0.306	−0.295	0.366	0.360	0.571	0.566	0.565	0.800	0.751	1.000	
−0.292	−0.305	−0.291	−0.281	0.348	0.342	0.542	0.537	0.537	0.760	0.714	0.646	1.000

Residual covariance matrix

Q1	Q2	Q3	Q4	Q5	Q6	Q7	Q8	Q9	Q10	Q11	Q12	Q13
< .001												
0.107	< .001											
< .001	< .001	< .001										
< .001	< .001	0.066	< .001									
0.060	0.032	< .001	< .001	< .001								
0.143	0.146	< .001	< .001	0.083	< .001							
0.118	0.091	< .001	< .001	0.031	0.029	< .001						
0.111	0.074	< .001	< .001	0.018	0.047	0.070	< .001					
0.101	0.068	< .001	< .001	< .001	< .001	< .001	< .001	< .001				
0.081	0.073	< .001	< .001	0.007	0.011	< .001	< .001	0.092	< .001			
0.075	0.067	< .001	< .001	< .001	< .001	< .001	< .001	0.056	0.005	< .001		
0.083	0.076	< .001	0.051	< .001	< .001	< .001	< .001	0.107	< .001	< .001	< .001	
0.070	0.064	0.002	0.023	< .001	< .001	< .001	< .001	0.063	< .001	< .001	0.115	< .001

Plots

Model plot

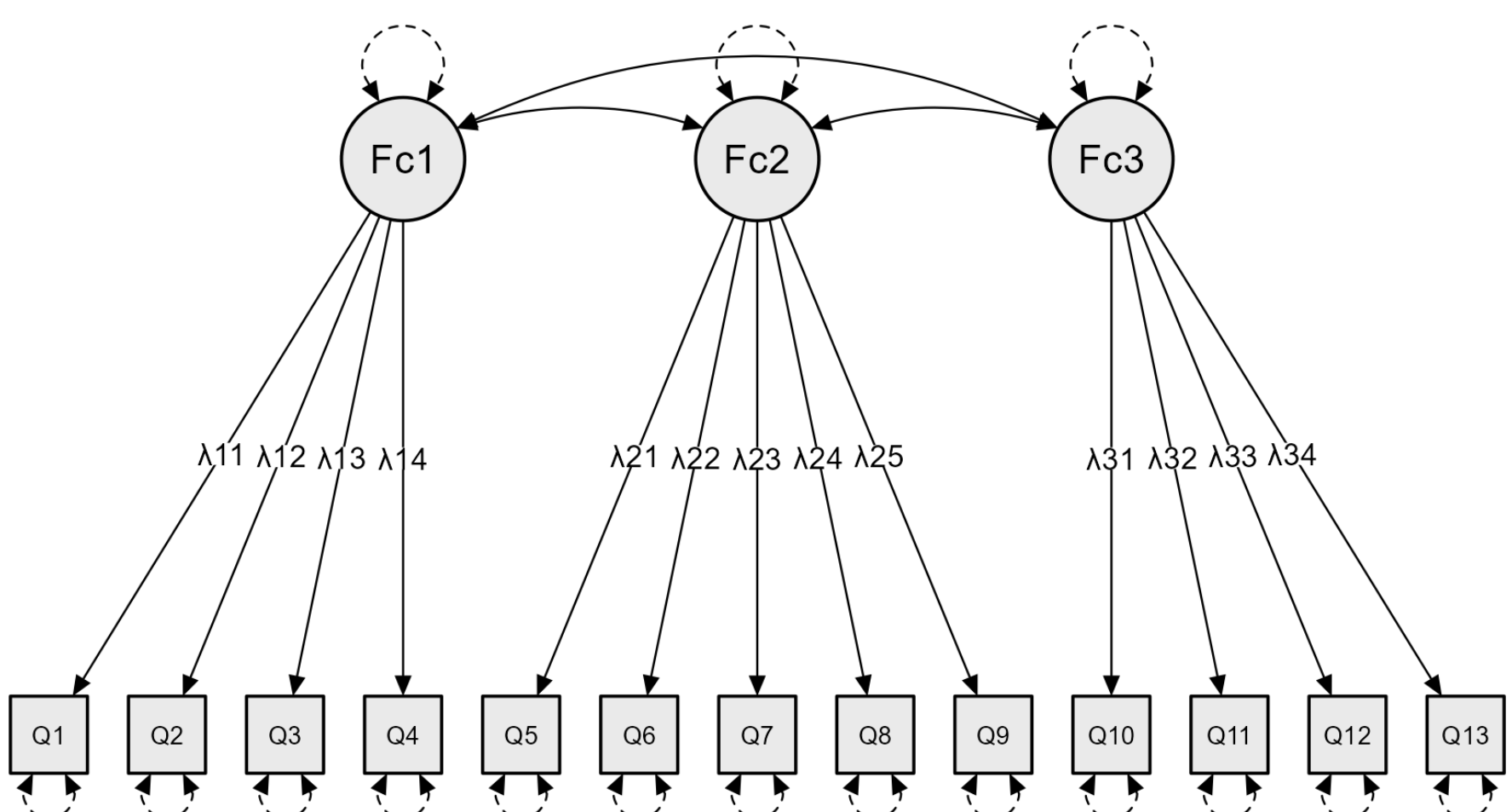

Supplement: Supplementary file 1 [file Data_Sheet_2.PDF]
