# Supplementary material for: Attitudes toward free speech, content moderation, and punishment of misinformation spreaders during infectious disease outbreaks
Source: Front Public Health. 2026 Jul 23;14:1824585. doi: 10.3389/fpubh.2026.1824585 (PMC13443021; doi:10.3389/fpubh.2026.1824585)
Supplement: Supplementary file 2 [file Data_Sheet_1.docx]

**Supplementary material to *Attitudes Toward Free Speech, Content Moderation, and Punishment of Misinformation Spreaders During Infectious Disease Outbreaks***

**The full dataset is available from the Jagiellonian University Repository in the #webimmunization collection: [https://ruj.uj.edu.pl/collections/d62d7240-967b-490d-b02b-e0402b1c506c](https://ruj.uj.edu.pl/collections/d62d7240-967b-490d-b02b-e0402b1c506c)**

**Social media, infectious diseases outbreak and free speech questionnaire**

Please provide us with your Prolific ID

**Instruction:**

Below, you will find several statements about free speech on social media platforms (such as X, formerly Twitter, Facebook, Bluesky, and Instagram) during outbreaks of infectious diseases (e.g., COVID-19, SARS, or a virus more virulent than the seasonal flu). People may have different opinions about these statements, with some agreeing and others disagreeing. Please rate how much you personally agree or disagree with each statement and how well it reflects your personal feelings or thoughts.

Use the following scale:

1 – Strongly disagree

2 – Disagree

3 – Somewhat disagree

4 – Neither agree nor disagree

5 – Somewhat agree

6 – Agree

7 – Strongly agree

For example, if you strongly disagree with a statement, select 1 – Strongly disagree. If you somewhat agree, select 5 – Somewhat agree.

After completing the questionnaire, you will be asked to provide demographic information, your political views and affiliation, and details about your social media use habits.

This research project does not collect or process any identifiable personal information.

This research is conducted by the Jagiellonian University Medical College. For inquiries, you can contact the principal investigator, Dr. Jan Piasecki, at webimmunization@uj.edu.pl.
1. Questionnaire

|  | Question | Strongly Disagree | Disagree | Somewhat Disagree | Neither agree nor disagree | Somewhat agree | Agree | Strongly  agree |
| --- | --- | --- | --- | --- | --- | --- | --- | --- |
| 1 | Every social media user has always a right to say whatever he/she wants. | 1 | 2 | 3 | 4 | 5 | 6 | 7 |
| 2 | Every social media user has always a right to share any kind of information. | 1 | 2 | 3 | 4 | 5 | 6 | 7 |
| 3 | Social media platforms should never censor any information created and shared by its users. | 1 | 2 | 3 | 4 | 5 | 6 | 7 |
| 4 | Social media platforms should never label any information created by its users. | 1 | 2 | 3 | 4 | 5 | 6 | 7 |
| 5 | Social media platforms may promote media literacy posts like this one: “Before you share, comment or like: make sure if this tweet is credible. Do you recognize the news organization/user that posted the story? Is information believable? Is this tweet politically motivated?” | 1 | 2 | 3 | 4 | 5 | 6 | 7 |
| 6 | During the COVID-19 outbreak tweets from public health departments, World Health Organization and infectious disease experts may be visible to every social media user, regardless of whether a user follows the account or not. | 1 | 2 | 3 | 4 | 5 | 6 | 7 |
| 7 | Posts that contain misinformation on an infectious diseases outbreak (as assessed by public health experts) may be tagged with a warning sign by social media platform (e.g. X, formerly Twitter, Facebook). | 1 | 2 | 3 | 4 | 5 | 6 | 7 |
| 8 | Posts containing misinformation on an infectious diseases outbreak may be accompanied with corrections from fact-checking organizations or public health departments. | 1 | 2 | 3 | 4 | 5 | 6 | 7 |
| 9 | Social media platforms should limit the possibility of reposting information about infectious diseases that is not supported by the current medical consensus. | 1 | 2 | 3 | 4 | 5 | 6 | 7 |
| 10 | Social media platforms should have a right to temporarily ban users who spread misinformation on an infectious disease outbreak. | 1 | 2 | 3 | 4 | 5 | 6 | 7 |
| 11 | Social media platforms should have a right to permanently ban users who spread misinformation on an infectious diseases outbreak (such as COVID-19). | 1 | 2 | 3 | 4 | 5 | 6 | 7 |
| 12 | The government should have a right to demand social media platforms to ban users spreading health misinformation during an infectious disease outbreak (such as COVID-19). | 1 | 2 | 3 | 4 | 5 | 6 | 7 |
| 13 | Spreading of health misinformation on social media should be a crime punishable with a fine | 1 | 2 | 3 | 4 | 5 | 6 | 7 |

**2. Demographic information**

Age:

Gender:

- Male
- Female
- Third gender / nonbinary / other

Employment:

Working full-time

Working part-time

Unemployed or looking for work

A homemaker or stay-at-home parent

Student

Retired

Other

Martial status

Married

Living with a partner

Widowed

Divorced/Separated

Never been married

Education:

Less than high school degree

High school graduate

Some college but not degree

Associate degree in college (2-year)

Bachelor's degree in college (4-year)

Master's degree

Doctoral degree

Political Affiliation

Republican

Democrat

Independent

Something else

Ethnicity:

White

Black or African American

American Indian or Alaska Native

Asian

Native Hawaiian or Pacific Islander

Other

**3. Political views**

Indicate your political orientation on a Scale from 1 (extremely liberal/left-wing), through 6 (middle of the road) to 11 (extremely conservative/right-wing)

**4. Social Media Usage Questionnaire**

1. How many hours do you spend on social media each day? (You can check it on your phone)

Less than 1 hour

1–2 hours

2–4 hours

4–6 hours

More than 6 hours

2. Which social media platforms do you use the most? (Select all that apply)

Facebook

Instagram

X (formerly Twitter)

Bluesky

TikTok

Snapchat

LinkedIn

Reddit

YouTube

Pinterest

Other

3. What do you primarily use social media for? (Select all that apply)

Networking

Business or professional purposes

Learning or educational content

Entertainment

Keeping in touch with friends and family

Sharing your thoughts, updates, or creations

Keep up with news

4. How many social media platforms do you actively use?

1

2

3

4

5

6 or more

5. How often do you use social media?

Multiple times a day

Daily

A few times a week

Weekly

Rarely

6. How often do you actively post on social media?

Very often (daily or more)

Often (several times a week)

Occasionally (a few times a month)

Rarely (less than once a month)

I don’t post

7. Is social media the first thing you check in the morning?

Yes

No

8. Would you describe your social media use as more active (e.g., posting, commenting, or creating content) or passive (e.g., scrolling, watching, or liking content)?

Mostly active

Mostly passive

A mix of both

Data analysis for the initial version of the COVID Questionnaire

1. The Questionnaire

|  | Question |
| --- | --- |
| 1 | Every Twitter user has always a right to say whatever he/she wants. |
| 2 | Every Twitter user has always a right to share any kind of information. |
| 3 | Twitter should never censor any information created and shared by its users. |
| 4 | Twitter should never label any information created and shared by its users. |
| 5 | Twitter should promote media literacy tweets like this one: “Before you share, comment or like: make sure if this tweet is credible. Do you recognize the news organization/user that posted the story? Is information believable? Is this tweet politically motivated?” |
| 6 | During the COVID-19 outbreak tweets from public health departments, World Health Organization and infectious disease experts should be visible to every Twitter user, regardless of whether a user follows the account or not. |
| 7 | Tweets that contain misinformation on COVID-19 outbreak (as assessed by public health experts) should be tagged with a warning sign by Twitter. |
| 8 | Tweets containing misinformation on COVID-19 outbreak should be accompanied with corrections from fact-checking organizations or public health departments. |
| 9 | Twitter should limit the possibility of retweeting information about COVID-19 that is not supported by the current medical consensus. |
| 10 | Twitter should have a right to temporarily ban users who spread misinformation on COVID-19 outbreak. |
| 11 | Twitter should have a right to permanently ban users who spread misinformation on COVID-19 outbreak. |
| 12 | The government should have a right to demand Twitter to ban users spreading health misinformation during the COVID-19 outbreak. |
| 13 | Spreading of health misinformation on Twitter should be a crime punishable with fine or imprisonment. |

2. Explanatory factor analysis

**Exploratory Factor Analysis**

| **Kaiser-Meyer-Olkin test** | | | |
| --- | --- | --- | --- |
|  | | **MSA** | |
| **Overall MSA** |  | **0.893** |  |
| **freespch_1** |  | **0.838** |  |
| **freespch_2** |  | **0.834** |  |
| **freespch_3** |  | **0.879** |  |
| **freespch_4** |  | **0.894** |  |
| **media_lit** |  | **0.953** |  |
| **health_edu** |  | **0.951** |  |
| **counter_1** |  | **0.893** |  |
| **counter_2** |  | **0.883** |  |
| **counter_3** |  | **0.957** |  |
| **counter_4** |  | **0.910** |  |
| **punish_5** |  | **0.899** |  |
| **punish_6** |  | **0.867** |  |
| **punish_7** |  | **0.863** |  |
|  | | | |

| **Bartlett's test** | | | | | |
| --- | --- | --- | --- | --- | --- |
| **Χ²** | | **df** | | **p** | |
| **28967.452** |  | **78.000** |  | **< .001** |  |
|  | | | | | |

| **Chi-squared Test** | | | | | | | |
| --- | --- | --- | --- | --- | --- | --- | --- |
|  | | **Value** | | **df** | | **p** | |
| **Model** |  | **1414.705** |  | **42** |  | **< .001** |  |
|  | | | | | | | |

| **Factor Loadings** | | | | | | | | | |
| --- | --- | --- | --- | --- | --- | --- | --- | --- | --- |
|  | | **Factor 1** | | **Factor 2** | | **Factor 3** | | **Uniqueness** | |
| **counter_2** |  | **0.920** |  |  |  |  |  | **0.167** |  |
| **counter_1** |  | **0.913** |  |  |  |  |  | **0.182** |  |
| **health_edu** |  | **0.709** |  |  |  |  |  | **0.567** |  |
| **media_lit** |  | **0.680** |  |  |  |  |  | **0.618** |  |
| **counter_3** |  | **0.561** |  |  |  |  |  | **0.329** |  |
| **freespch_2** |  |  |  | **0.941** |  |  |  | **0.195** |  |
| **freespch_1** |  |  |  | **0.900** |  |  |  | **0.274** |  |
| **freespch_3** |  |  |  | **0.872** |  |  |  | **0.229** |  |
| **freespch_4** |  |  |  | **0.762** |  |  |  | **0.361** |  |
| **punish_6** |  |  |  |  |  | **0.925** |  | **0.267** |  |
| **punish_7** |  |  |  |  |  | **0.884** |  | **0.376** |  |
| **punish_5** |  |  |  |  |  | **0.655** |  | **0.284** |  |
| **counter_4** |  |  |  |  |  | **0.494** |  | **0.248** |  |
|  | | | | | | | | | |
| ***Note.*  Applied rotation method is promax.** | | | | | | | | | |

| **Factor Loadings (Structure Matrix)** | | | | | | | |
| --- | --- | --- | --- | --- | --- | --- | --- |
|  | | **Factor 1** | | **Factor 2** | | **Factor 3** | |
| **freespch_1** |  |  |  | **0.843** |  |  |  |
| **freespch_2** |  |  |  | **0.892** |  |  |  |
| **freespch_3** |  | **-0.408** |  | **0.878** |  |  |  |
| **freespch_4** |  | **-0.424** |  | **0.792** |  |  |  |
| **media_lit** |  | **0.614** |  |  |  |  |  |
| **health_edu** |  | **0.647** |  |  |  | **0.405** |  |
| **counter_1** |  | **0.902** |  | **-0.453** |  | **0.551** |  |
| **counter_2** |  | **0.911** |  | **-0.438** |  | **0.569** |  |
| **counter_3** |  | **0.780** |  |  |  | **0.695** |  |
| **counter_4** |  | **0.759** |  | **-0.510** |  | **0.792** |  |
| **punish_5** |  | **0.656** |  | **-0.473** |  | **0.818** |  |
| **punish_6** |  | **0.493** |  |  |  | **0.850** |  |
| **punish_7** |  | **0.421** |  |  |  | **0.778** |  |
|  | | | | | | | |
| ***Note.*  Applied rotation method is promax.** | | | | | | | |

| **Factor Characteristics** | | | | | | | | | | | | | |
| --- | --- | --- | --- | --- | --- | --- | --- | --- | --- | --- | --- | --- | --- |
|  | | **Unrotated solution** | | | | | | **Rotated solution** | | | | | |
|  | | **SumSq. Loadings** | | **Proportion var.** | | **Cumulative** | | **SumSq. Loadings** | | **Proportion var.** | | **Cumulative** | |
| **Factor 1** |  | **5.945** |  | **0.457** |  | **0.457** |  | **3.282** |  | **0.252** |  | **0.252** |  |
| **Factor 2** |  | **2.042** |  | **0.157** |  | **0.614** |  | **3.114** |  | **0.240** |  | **0.492** |  |
| **Factor 3** |  | **0.916** |  | **0.070** |  | **0.685** |  | **2.507** |  | **0.193** |  | **0.685** |  |
|  | | | | | | | | | | | | | |

| **Factor Correlations** | | | | | | | |
| --- | --- | --- | --- | --- | --- | --- | --- |
|  | | **Factor 1** | | **Factor 2** | | **Factor 3** | |
| **Factor 1** |  | **1.000** |  | **-0.447** |  | **0.648** |  |
| **Factor 2** |  | **-0.447** |  | **1.000** |  | **-0.372** |  |
| **Factor 3** |  | **0.648** |  | **-0.372** |  | **1.000** |  |
|  | | | | | | | |

| **Additional fit indices** | | | | | | | | | | | |
| --- | --- | --- | --- | --- | --- | --- | --- | --- | --- | --- | --- |
| **RMSEA** | | **RMSEA 90% confidence** | | **SRMR** | | **TLI** | | **CFI** | | **BIC** | |
| **0.104** |  | **0.1 - 0.109** |  | **0.021** |  | **0.912** |  | **0.952** |  | **1078.480** |  |
|  | | | | | | | | | | | |
